# Effects of Ultrasound-Assisted Extraction on Structure and Rheological Properties of Flaxseed Gum

**DOI:** 10.3390/gels9040318

**Published:** 2023-04-10

**Authors:** Xuejiao Ren, Xin Meng, Zhen Zhang, Hongyu Du, Tuoping Li, Na Wang

**Affiliations:** 1 College of Food and Health, Jinzhou Medical University, Jinzhou 121000, China; applei3@163.com (X.R.);; 2 Innovation Center of Meat Processing and Quality Control Technology of Liaoning Province, Jinzhou Medical University, Jinzhou 121000, China; 3 College of Food, Shenyang Agricultural University, Shenyang 110866, China; 4 Department of Pathology, School of Basic Medical Sciences, Jinzhou Medical University, Jinzhou 121000, China

**Keywords:** flaxseed gum, ultrasound-assisted extraction, structural properties, rheological properties

## Abstract

In this study, flaxseed gum (FG) was extracted using hot water extraction and ultrasonic-assisted extraction. The yield, molecular weight distribution, monosaccharide composition, structure, and rheological properties of FG were analyzed. The FG yield (9.18) achieved using ultrasound-assisted extraction (this sample was labeled as UAE) was higher than the yield (7.16) achieved with hot water extraction (this sample was labeled as HWE). The polydispersity, monosaccharide composition, and characteristic absorption peaks of the UAE were similar to that of the HWE. However, the UAE had a lower molecular weight and looser structure than the HWE. Moreover, zeta potential measurements indicated that the UAE exhibited better stability. An analysis of the rheological properties showed that the viscosity of the UAE was lower. Thus, the UAE had an effectively better yield of FG, preliminarily modified structure, and rheological properties, and provided a theoretical basis for its application in food processing.

## 1. Introduction

Food hydrocolloids are cheap and safe hydrophilic substances that can be added to food for improving water absorption, water retention, emulsion stability, and rheology [1].

Flaxseed gum (FG) is a by-product of flaxseed oil production, and is mostly found in the outer layer of flaxseed. Aqueous solutions of FG have been shown to exhibit several desirable functional properties and, therefore, have attracted significant research interest [2]. In the food industry, FG can be used as a thickening agent, stabilizer, and emulsifier. Furthermore, it was shown to have good water retention, rheological, and gelling properties.

FG lowers blood sugar and cholesterol owing to the reduction in carbohydrate absorption after the consumption of FG. Thakur et al. [3] mixed FG into wheat flour pancakes for patients with type 2 diabetes for 60 days, which reduced the LDL cholesterol from 1.11 × 10^4^ mg/dL to 9.30 × 10^3^ mg/dL. This study confirmed the efficacy of FG in type 2 diabetes. The FG solution has a high viscosity. Liu et al. [4] prepared FG from six Canadian linseed varieties, in which the highest solution viscosity was 2.984 Pa·s and the lowest was 0.048 Pa·s, with significant differences between different varieties. Adding NaCl decreases the solution’s viscosity, while adding sucrose increases the solution’s viscosity. FG has properties similar to that of “weak gels” and can be used to replace most nongels in food and nonfood applications. Chen et al. [5] studied the FG formation conditions and the factors influencing gel strength; they found that FG is formed as a hot reversible gel. The increase in the gel’s strength was positively correlated with the temperature. Under a pH of 6–9, the maximum gel strength could be obtained, the addition of sodium ions reduced the gel’s strength, and the low concentration of calcium ions could increase the gel’s strength. Safdar et al. [6] compared four FG extraction methods. The FG extracted using the four methods showed concentration dependence in the antioxidant test, among which the ultrasound-assisted FG exhibited a higher radical removal ability of ABTS, DPPH, reduction, and β-carotene. Chen et al. [7] and others obtained flaxseed polysaccharides by grading FG. Flaxseed polysaccharides showed good antioxidant activity in hydroxyl, ABTS, and DPPH radicals. An in vitro cytotoxicity test showed that flaxseed polysaccharides had a dose-dependent ability to inhibit the proliferation of HepG 2 and HeLa cells.

Currently, the extraction processes for FG primarily include hot water extraction, microwave-assisted extraction [8], ultrasound-assisted extraction [9], and enzyme-assisted extraction [10]. Several new processes, such as supercritical extraction [11], ionic liquid extraction [12], ultra-high-pressure extraction [13], rapid solvent extraction, and subcritical water extraction, have also been developed [11]. The choice of extraction method can affect the extraction rate of the polysaccharides, as well as their structure and functional properties. Hot water extraction is a conventional polysaccharide extraction method that has the advantages of requiring simple equipment, ease of operation, and low cost. However, it has many disadvantages, such as a long extraction time, high temperature, and low efficiency. Ultrasound-assisted extraction mainly uses the cavitation effect of ultrasound waves, which destroy the plant cell wall through mechanical and thermal effects, thereby promoting the dissolution and diffusion of the polysaccharides and improving the extraction rate. Hence, it is used widely for polysaccharide extraction.

Presently, the domestic application of ultrasound-assisted extraction mainly focuses on the extraction process, and research on the structural and functional properties of the as-extracted FG samples is lacking. In this study, an ultrasound-assisted process was employed to extract FG from flaxseed. Furthermore, its chemical structure was characterized and rheological properties were analyzed.

## 2. Results and Discussion

### 2.1. FG Yield

As a traditional extraction method, the hot water extraction method is inexpensive, can avoid contamination caused by chemical solvents, and has high safety. Under the conditions of hot water extraction, the yield of the HWE was 7.16% ± 0.08. However, the yield of the UAE could reach 9.18% ± 0.20. Morales et al. compared the yield of chan seed mucilage with different methods and obtained the same conclusion [14]. These results were consistent with those reported by Farbe et al. [15] with camelina seed mucilage and Kia et al. [16] with fenugreek seed gum. Ultrasound-assisted extraction mainly uses the cavitation effect of ultrasonic waves to destroy the plant cell wall due to mechanical and thermal effects, thereby promoting the dissolution and diffusion of gum and improving its extraction rate. Ultrasound-assisted extraction shortens the extraction time and improves the extraction rate of FG (*p* < 0); therefore, it is an efficient, low-temperature, short-duration method suitable for extracting polysaccharide substances such as FG.

### 2.2. Molecular Weight Analysis of FG

The Mw of polysaccharides reflects the characteristics of their molecular chain, which, in turn, affect their bioactivity [17]. When the molecular weight of certain polysaccharides decreases, their intrinsic viscosity decreases, whereas their water solubility and biological activity increase [18,19]. However, this may result in a reduction in their biological activity. Therefore, investigating the molecular weight of FG is important. The type of glycosidic bonds, monosaccharide composition [20], and mode of connection of the polysaccharides are not related to their natural properties.

**Figure 1:**
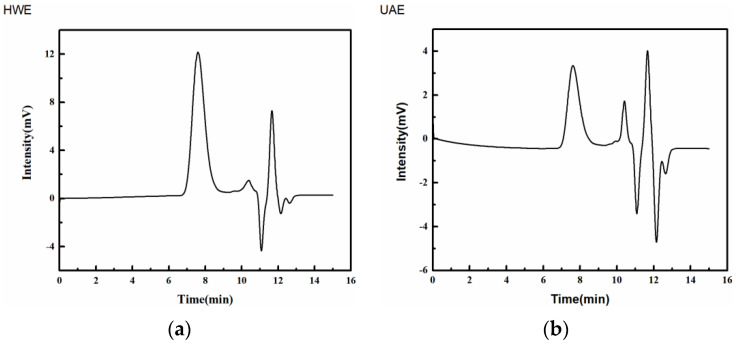
GPC chromatogram of HWE and UAE: (**a**)—HWE, (**b**)—UAE.

The Mw, number average mass (M_n_), and polydispersity index (PDI) values of the FG samples extracted using the two methods are listed in Table 1. Based on the elution curve in Figure 1, the samples exhibited multiple peaks during the high-performance gel-permeation chromatography (HP-GPC), indicating that both samples were heteropolysaccharides. The molecular masses of the FG samples were 770 (HWE) and 767 kDa (UAE). The Mw of the UAE was slightly lower than that of the HWE. This was due to the ultrasonic cavitation effect, which helped dissolve the polysaccharide gum into the extraction solvent [21] by destroying its molecular chain [22] and decomposing the polysaccharide molecules into smaller ones. However, the Mw value of the FG in this study was lower than that of the FG 1.6 × 10^6^ g/mol (1600 kDa) − 10 × 10^6^ g/mol (10,000 kDa) reported by Roulard et al. [23]. The difference in the mean molecular weight may have been due to differences in the plant growth areas, varieties, extraction methods, purification methods, and analytical procedures [24].

The M_w_/M_n_ ratio is used to measure the width of a polymer and determine its molecular weight distribution. The larger the PDI value, the wider the Mw distribution. A PDI value of 1 indicates that the system has a uniform Mw distribution. The PDI values of both the HWE and UAE samples were 1.11, indicating that the FG samples extracted using the two methods had a relatively uniform molecular size, narrow molecular weight distribution, and concentrated molecular distribution. This suggested that the extraction method used did not change the nature of the polysaccharides, but could destroy their skeleton, leading to a decrease in their Mw [21].

### 2.3. Analysis of the Monosaccharide Composition

**Figure 2:**
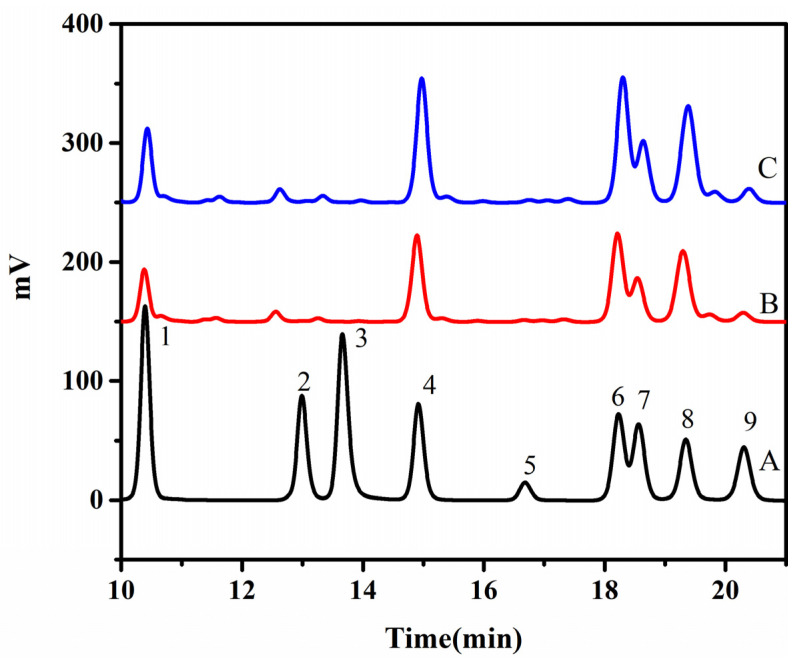
Monosaccharide compositions of FG samples obtained using different extraction methods: A—monosaccharide standard; B—HWE; C—UAE.

The composition of monosaccharides determines the structure and properties of the polysaccharide gum [25]. The monosaccharide compositions of the FG samples were determined via HPLC based on the retention time for the standard sample, with the results presented in Figure 2. The monosaccharide compositions of the obtained HWE and UAE samples were the same. The content of each monosaccharide is listed in Table 2. The FG samples were mainly composed of arabinose, xylose, galacturonic acid, galactose, rhamnose, fucose, and glucose. The peak areas of the HPLC curves were used to calculate the specific content of each monosaccharide. In both the HWE and UAE samples, the arabinose content was the highest, followed by those of xylose and galacturonic acid. A few differences were observed between the two samples. Therefore, the results showed that the FG was a heteropolysaccharide, mainly composed of neutral and acidic monosaccharides. The neutral monosaccharides were mainly xylose and arabinose, with traces of galactose and glucose [26], whereas the acidic monosaccharides were primarily rhamnose, fucose, and fructose [27].

The analysis also confirmed that different extraction methods had no effect on the monosaccharide composition of FG, but did change the contents of different monosaccharides. The monosaccharide composition is a natural characteristic of polysaccharides and is not affected by the extraction method [28]. He et al. [25] used hot water extraction, ultrasound-assisted extraction, and microwave-assisted extraction to extract polysaccharides from dandelion stems and reached a similar conclusion. The overall monosaccharide composition of the FG samples analyzed in this study was consistent with those reported previously [29]. However, the contents of the different detected monosaccharides varied, owing to the differences in flaxseed variety, extraction conditions, and extraction process [27].

### 2.4. FT-IR Analysis

**Figure 3:**
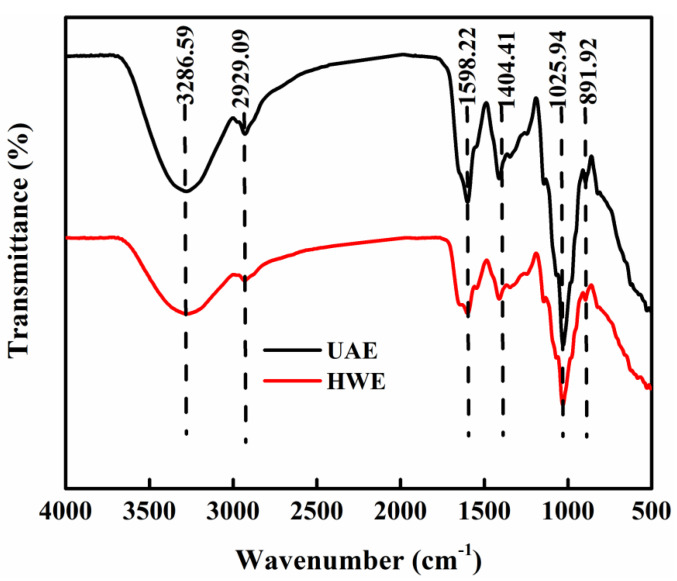
FT–IR spectra of FG samples extracted using different methods.

The FT-IR profiles of the HWE and UAE (Figure 3) comprised a characteristic polysaccharide absorption peak in the 400–4000 cm^−1^ range. The strong and broad absorption peak observed at 3278.73 cm^−1^ was related to the tensile vibrations of the hydroxyl (–OH) group [30], which forms hydrogen bonds [31]. The weak absorption peak observed at 2929.08 cm^−1^ was due to the tensile vibrations of the hydrocarbon group (C–H) [32]. The characteristic peaks at 1599.77 and 1411.31 cm^−1^ correlated with the vibration of the carboxylic acid (–COO) group [33], indicating the presence of glucuronic acid in the FG samples. The region between 800 and 1200 cm^−1^ is the fingerprint region, and if there was a difference in the structure of the compounds, their absorption peaks in the fingerprint region would have to be different [34]. The series of peaks in the 1000–1200 cm^−1^ region was confirmed to be typical of polysaccharides [35]. The peak at 1148.70 cm^−1^ indicated the presence of C–OC–C and C–OH glycosidic bonds, whereas the peak at 1028.92 cm^−1^ indicated the presence of glucan units or was related to the tensile vibrations of the pyranose ring. The weak absorption band at 895.02 cm^−1^ was related to the bending vibrations of the C–H bond and indicated the presence of a β-configuration in the FG structure. In conclusion, the FT-IR spectra of the FG samples contained typical polysaccharide-related absorption peaks and confirmed that the UAE-FG retained the polysaccharide structure of the FG.

Safdar et al. also performed an FT-IR analysis of FG [6] and found bands at 3400, 2934, 1610, 1415, 1145, 826.9, 629.5, and 534.7 cm^−1^. The FG samples extracted in this study were different from those reported in the literature, owing to the differences in their varieties and monosaccharide compositions [36].

### 2.5. SEM Analysis

**Figure 4:**
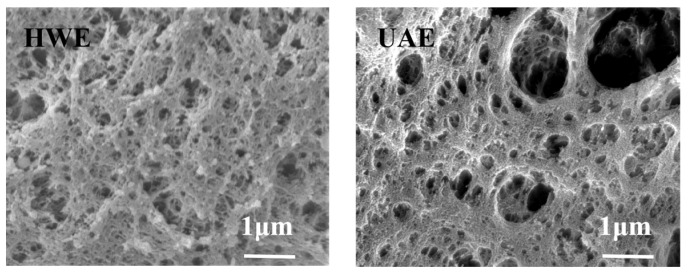
SEM images of FG samples extracted using different methods.

The surface morphologies of the FG samples were studied using SEM (Figure 4). Previously, the HWE presented a dense honeycomb structure. After the ultrasonic treatment, the surface morphology of the UAE underwent obvious changes, and the tissue structure changed. The structure became loose and holes of various sizes appeared. This difference in microstructures was because of the cavitation effect of the ultrasound waves, which disrupted the intermolecular bonds of the FG and reorganized its intermolecular network structure.

### 2.6. Zeta Potential Measurements

**Figure 5:**
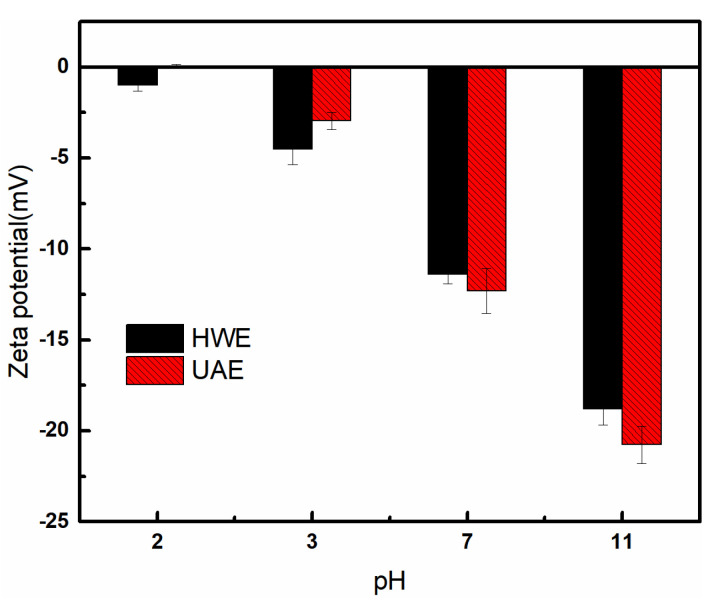
Zeta potentials of HWE and UAE samples at different pH values.

When the zeta potential is high, the dispersed particles have a higher tendency to repel each other. This prevents them from coming close to each other and forming aggregates. The changes in the zeta potential of FG in the 2.0–11.0 pH range are shown in Figure 5. At pH = 2.0, the zeta potential of the HWE sample was −1.00 mV, and that of the UAE was 0.04 mV. Both values were close to zero, indicating that the surface charge of the FG was remarkably close to zero, which was consistent with the results reported by Vieira [37]. When the pH value ranged from 3.0 to 11.0, the potential of the FG was negative. However, the absolute value increased with the increasing pH value. The isoelectric point of the isolated flaxseed protein was at pH 4.2 [38]. However, it was not observed in this study, owing to the low protein content of the extracted FG samples. When the pH was increased from 2.0 to 6.0, the absolute value of the potential of the HWE sample was higher than that of the UAE, and when the pH was between 6.0 and 11.0, the absolute value of the potential of the HWE sample decreased. The extraction of FG mainly occurred in a neutral environment (pH of approximately 7.0), and the zeta potential of the HWE sample was less than that of the UAE. The reason for this may have been the difference in the monosaccharide compositions of the two FG samples.

### 2.7. XRD Analysis

**Figure 6:**
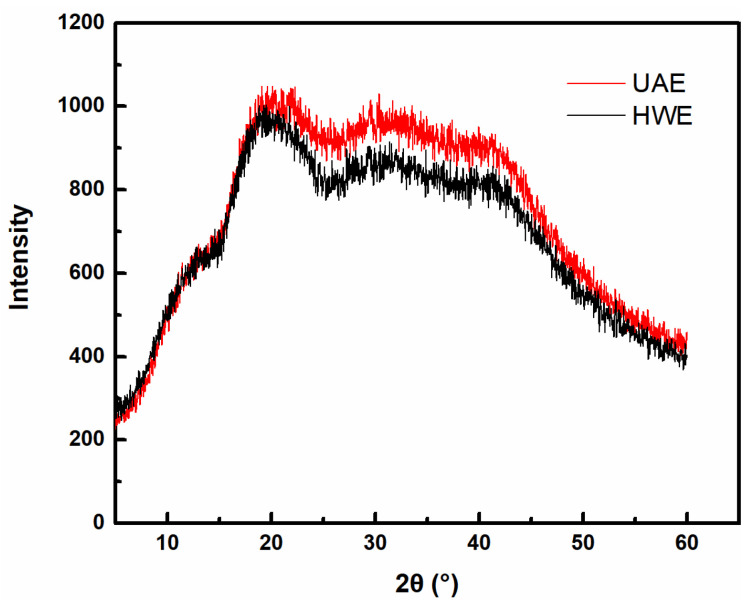
XRD patterns of FG samples extracted using different methods.

An XRD analysis was performed to evaluate the structures of the FG samples without crystallization regions. In the carbohydrate-based FG, an abundance of OH groups led to the formation of intra- and intermolecular hydrogen bonds, which, in turn, led to varying degrees of junction coherence and an amorphous transformation. The XRD profiles of the HWE and UAE samples are shown in Figure 6. The HWE sample exhibited a broad peak at 2θ = 19.2°, which was attributable to electrostatic interactions and indicated that the sample was noncrystalline. This result was consistent with the findings of Devi et al. [39], who observed that flaxseed mucus was a typical amorphous substance that exhibited a broad and low-intensity XRD peak, which indicated a lack of crystallinity or an ordered structure. The UAE sample exhibited a similar XRD pattern; however, its peak was observed at a relatively higher 2θ value, and the peak area was smaller, indicating a change in the crystal structure of the UAE and, hence, its XRD pattern. This may have been related to the low molecular weight of this sample. The amorphous nature of the polysaccharides resulted in a high kinetic solubility and a high dissolution rate.

### 2.8. Analysis of Rheological Properties of FG

#### 2.8.1. Static Rheological Properties

**Figure 7:**
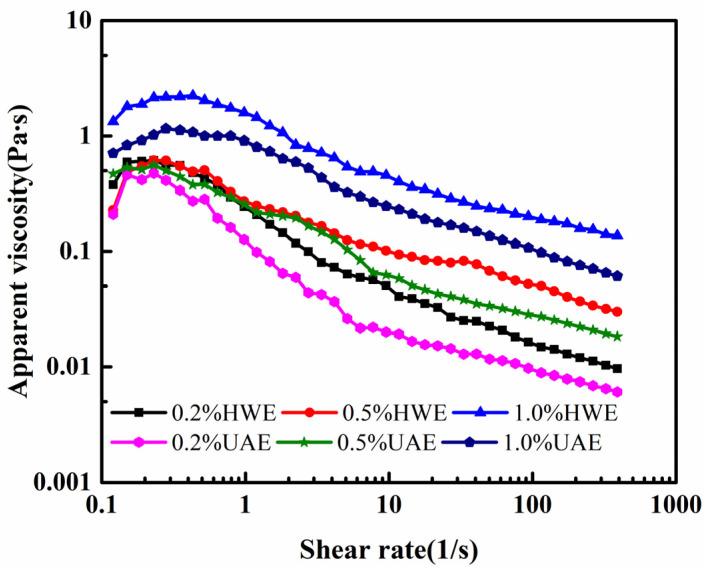
Apparent viscosity curves of FG at different concentrations.

The static rheological curves of the HWE and UAE are shown in Figure 7. The shear rate gradually increased from 0.1 to approximately 400 s^−1^. As the sample concentration increased, its apparent viscosity increased. This may have been due to the entanglement and aggregation of the molecular chains at higher concentrations [40]. Moreover, the viscosity of the FG solution decreased with an increasing shear rate for all three concentrations, indicating a non-Newtonian fluid. This result was consistent with the results reported by Wang [41]. The results showed that the apparent viscosity of the UAE was low, which may have been because the ultrasound-assisted extraction destroyed the association between the molecular chains, increased the relative movement of the molecular chains, and reduced the viscosity of the solution.

#### 2.8.2. Dynamic Rheological Properties

The interaction patterns of the polysaccharide molecules could be inferred based on their rheological properties [42]. The dynamic rheological characteristics of the materials are generally expressed in terms of G′ and G″. Previous studies have shown that FG shows weak gel-like characteristics [29]. To investigate the dynamic rheological characteristics of FG, the correlation between its G′ and G″ values and the frequency was determined under constant stress.

**Figure 8:**
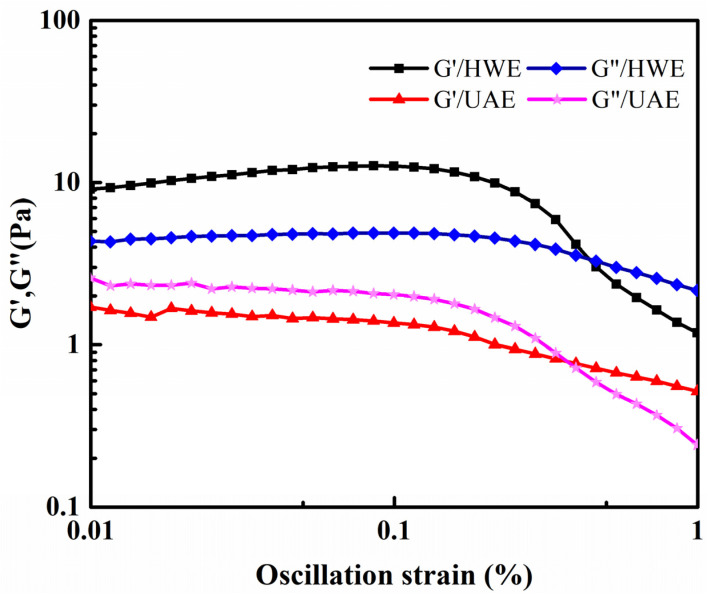
Results of strain sweeps of FG samples.

To investigate the dynamic rheological properties of the HWE and UAE samples, solutions with a concentration of 0.5% (*w*/*v*) were used. To determine the linear viscoelastic region, the first sweep of the solution was performed at a fixed frequency of 1 Hz (Figure 8). The samples were measured immediately without preshearing. The G′ and G″ values remained essentially constant when the strain was less than 0.1% and within the linear region. For frequency scanning, a strain of 0.1% was used.

**Figure 9:**
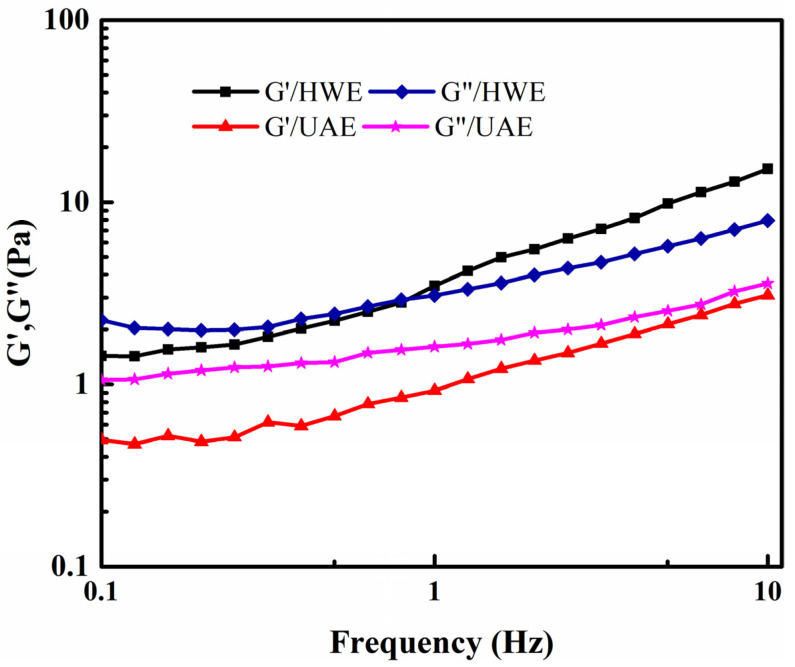
Scanning frequency curves of FG samples produced using different methods.

The dynamic rheological curves of the HWE and UAE samples are shown in Figure 9; G′ and G″ both increased with the increasing frequency. Moreover, with the increasing frequency, G′ was higher than G″; this is indicative of weak gel-like properties. Cui [43] reported that arabinoxylan had a determining effect on the shear dilution and weak gel-like characteristics of FG, such that FG samples containing more neutral monosaccharides exhibited a stronger shear dilution behavior and weaker gel-like characteristics, whereas FG samples containing more acidic monosaccharides (rhamnose and galacturonic acid) showed rheological characteristics typical of viscoelastic fluids. Therefore, the FG samples were non-Newtonian fluids with weak gel-like characteristics.

## 3. Conclusions

Compared with traditional hot water extraction, the ultrasonic-assisted extraction method significantly improved the yield and slightly decreased the molecular weight of the FG. There was no significant differences in the monosaccharide composition between the HWE and UAE, and both contained arabinose, xylose, and galacturonic acid. The ultrasound-assisted extraction altered the contents of various monosaccharides; the UAE had less arabinose and xylose, and more galacturonic acid. The FT-IR analysis showed that both FG samples exhibited the same characteristic absorption peaks. The SEM and XRD analyses showed that the UAE had a looser structure, indicating that the ultrasound-assisted extraction method changed the original structure of the FG. The zeta potential measurements showed that the UAE exhibited a high stability. The ultrasound-assisted extraction reduced the viscosity and elasticity of FG, resulting in more fluidic behavior of the FG solutions.

## 4. Materials and Methods

### 4.1. Materials

Flaxseed was purchased from a local market in Wulanchabu, Inner Mongolia. Ethanol and trifluoroacetic acid (TFA) were of analytical reagent grade (≥99.5%) and purchased from Sinopharm Chemical Reagent Co., Ltd. (Shanghai, China). The sugar was of analytical standard and provided by Aladdin (Shanghai, China). Ultrapure (UP) water (18.2 MΩ·cm, 25 °C) was obtained using a purification system supplied by Millipore, Bedford, MA, USA.

### 4.2. Preparation of FG Samples

Hot water extraction method: First, 100 g of raw flaxseed was weighed in a beaker, and UP water was added at a liquid ratio of 1:15 (g/mL). The solution was then centrifuged at 5000 r/min for 15 min after continuously stirring in an 80 °C water bath for 2 h. Next, three volumes of 95% ethanol were added to the supernatant. The resulting solution was allowed to stand and was then centrifuged at 5000 r/min for 15 min. Finally, the precipitate, labeled as HWE, was dried at 50 °C.

Ultrasound-assisted extraction method: First, 100 g of raw flaxseed was weighed in a beaker, and UP water was added in a liquid ratio of 1:15 (g/mL). The mixture was treated at 60 °C and 240 W for 30 min with ultrasound (KQ-300GDV, Kun Shan Ultrasonic Instruments Co., Ltd., Jiangsu, China). The resulting solution was centrifuged at 5000 r/min for 15 min. Three volumes of 95% ethanol were added to the supernatant, which was soaked overnight and then centrifuged at 5000 r/min for 15 min. Finally, the precipitate, labeled as UAE, was dried at 50 °C.

### 4.3. Determination of FG Yield

The yield of FG was calculated using the following formula:

$$\mathrm{Yield}\;(\%)=\frac{w_{g}}{w_{s}}\times 100$$

where *W_g_* is the weight of the HWE or UAE (*g*) and *Ws* is the weight of the flaxseed (*g*) for extraction [6].

### 4.4. Characterization Tests

#### 4.4.1. Determination of Molecular Weights and Molecular Weight Distributions of FG Samples

Predetermined amounts (1–2 mg) of the FG samples (HWE and UAE) were dissolved in deionized water at concentrations of 2 mg/mL, and the solutions were centrifuged using a 0.22 μm filter. The molecular weight (Mw) was investigated using gel permeation chromatography (Waters 1525-2414, American Waters, Inc., New Port Richey, FL, USA). The column temperature was 35 °C, the loading volume was 20 μL, and the flow rate was 1.0 mL/min.

#### 4.4.2. Determination of the Monosaccharide Composition

Sample processing: Predetermined amounts (10.0 mg) of the FG samples were hydrolyzed using 5 mL of a 2 mol/L TFA solution at 80 °C for 16 h. The samples were cooled and then hydrolyzed again at 100 °C for 1 h using 5 mL of 2 mol/L TFA. After cooling, the TFA was removed using 1 mL of 1 mol/L methanol and a 70 °C water bath in a N_2_ atmosphere. Next, 1 mL of a 0.3 mol/L NaOH solution was added to the samples.

Monosaccharide derivatization: A predetermined amount (0.4 mL) of a monosaccharide standard solution or polysaccharide hydrolysate in a 5 mL plug test tube was added to 0.4 mL of a 1-phenyl-3-methyl-5-pyrazolone-methanol solution. The mixture was vortex-mixed and reacted in a 70 °C water bath for 2 h. The solution was removed and allowed to cool to 20 °C. Next, 0.4 mL of 0.3 mol/L HCl was added for neutralization, followed by 1.2 mL UP water and an equal volume of chloroform. This mixture was also vortex-mixed, shaken, and left to stand. The chloroform phase was discarded. The aqueous phase was then filtered using a 0.45 μm microporous membrane (drainage) for the high-performance liquid chromatography (HPLC) injection analysis.

Chromatographic conditions (HPLC Agilent1100 was equipped with a diode array detector, American Agilent, Inc., Santa Clara, CA, USA): Column C18, 250 mm 4.6 mm; particle size, 5 μm; mobile phase A: 90 mmol/L sodium phosphate buffer (pH = 7.8); mobile phase B: acetonitrile; detection wavelength: 250 nm; column temperature, 30 °C; flow rate, 1 mL/min; sample volume, 10 μL. The gradient elution conditions are listed in Table 3.

#### 4.4.3. Fourier-Transform Infrared (FT-IR) Spectral Analysis

The infrared absorption spectra of the FG samples were measured using the KBr pressure tablet method with an FT-IR spectrum (Nicolet is, Thermo Fisher Scientific, Waltham, MA, USA) analyzer over the scanning interval of 400–4000 cm^−1^. A total of 32 scans were performed. The OMNIC software supplied with the instrument was used to analyze the spectra [6].

#### 4.4.4. Scanning Electron Microscopy (SEM) Analysis

The morphologies of the samples were observed with SEM (GeminiSEM 500, ZEISS, Jena, Germany). A small amount of the FG sample powder to be tested was pasted on the conductive adhesive tape of the sample table. The impurities were gently blown away with an ear-washing ball, and the surface characteristics of the sample were observed at an accelerating voltage of 5 kV.

#### 4.4.5. Zeta Potential Measurements

The FG powder to be tested was dispersed in UP water at a concentration of 0.1% (*w*/*v*) and filtered using filter paper. The dispersed FG was then transferred to a glass tube and diluted 100 times with deionized water to avoid multiple scatterings. The pH of the diluted sample was adjusted to 2.0–11.0 with 0.1 M HCl or 0.1 M NaOH. The zeta potential of the FG solution under different pH conditions was determined thrice for each sample, and the values were averaged (Zetasizer Nano ZS-90, Malvern Instruments, Malvern, UK) [44].

#### 4.4.6. X-ray Diffraction (XRD) Analysis

The crystal structures of the FG samples were determined using an XRD analysis system (D8 Advance, Bruker, Billerica, MA, USA) with a Cu target/Cu-Kα source (1.542 Å) at a voltage of 40 kV and current of 40 mA. The scan range (2θ) was 3°–60°, the step size was 0.02, and the scan speed was 4 min^−1^.

#### 4.4.7. Determination of Rheological Properties

Determination of apparent viscosity (η): The FG sample was dissolved in deionized water in concentrations of 0.2%, 0.5%, and 1.0% (*w*/*v*) using a 60 °C water bath for 2 h. A probe with a flat circular smooth plate (60 mm diameter) and a clearance of 1.0 mm was used to measure the apparent viscosity of the sample and to determine its apparent viscosity curve (Rheometer MCR 92, Anton paar, Austria Co. Ltd., Graz, Austria); the measurements were performed by increasing the shear rate from 0.1 to 400 s^−1^ at 25 °C.

Determination of viscoelastic properties: The viscoelastic properties of the FG samples were determined with dynamic oscillation scanning, which was performed using a flat circular steel plate (40 mm in diameter), and the storage modulus (G′) was evaluated at a certain concentration (0.5%, *w*/*v*). The loss modulus (G″) was determined through scanning at a frequency of 0.1–10 Hz and a temperature of 25 °C. Stress scans were performed at a constant oscillating frequency of 1 Hz to determine the linear viscoelastic regions before the frequency scans. All the frequency scans were performed at a strain value of 0.1%.

### 4.5. Statistical Analysis

The variance and its significance were analyzed using the SPSS Statistics 22.0 software (IBM, Armonk, NY, USA) and Origin 2016 software (OriginLab, Northampton, MA, USA), and the data were analyzed for the differential significances using the analysis of variance technique. All the data used were the averages obtained from parallel experiments.

## Figures and Tables

**Table 1:** Molecular weights of various FG samples.

Sample	M_n_ (Da)	Mw (Da)	M_z_ (Da)	M_z_ + 1 (Da)	Polydispersity Index (PDI)	M_z_/M_w_	M_z_ + 1/M_w_
HWE	6.95 × 10^5^	7.70 × 10^5^	8.21 × 10^5^	8.58 × 10^5^	1.11	1.07	1.11
UAE	6.94 × 10^5^	7.67 × 10^5^	8.18 × 10^5^	8.55 × 10^5^	1.11	1.07	1.15

**Table 2:** Monosaccharide contents of FG samples obtained using different extraction methods.

	Monosaccharide Content (mg/mL)	HWE	UAE
1	Rhamnose	6.18	6.20
2	Glucuronic acid	-	-
3	Aminogalactose	-	-
4	Galacturonic acid	21.20	21.65
5	Glucose	2.95	3.07
6	Xylopyranose	23.73	23.54
7	Galactose	12.78	12.61
8	Arabinose	28.70	28.20
9	Fucose	4.07	4.20

**Table 3:** Gradient elution conditions.

T (min)	A%	B%
0	86	14
9	83	17
28	78	22
29	50	50
31	50	50
32	86	14
36	86	14

## Data Availability

Data available on request from the authors.
